# A Case of Congenital Brainstem Oligodendroglioma: Pathology Findings and Review of the Literature

**DOI:** 10.1155/2017/2465681

**Published:** 2017-07-26

**Authors:** Stefan Kostadinov, Suzanne de la Monte

**Affiliations:** ^1^Department of Pathology and Laboratory Medicine, Women & Infants Hospital, 101 Dudley Street, Providence, RI, USA; ^2^The Alpert Medical School of Brown University, Providence, RI, USA; ^3^Divisions of Neuropathology and Neurosurgery, Rhode Island Hospital, 593 Eddy Street, Providence, RI 02903, USA

## Abstract

Congenital and perinatal primary brain neoplasms are extremely rare. Brainstem neoplasms in the perinatal and neonatal period are typically of high-grade nature and have poor prognoses with survival rates of less than 2 years from diagnosis. Herein, we report an unusual case of congenital anaplastic oligodendroglioma that arose in the pons and was detected as diffuse pontine glioma on in utero imaging studies during prenatal evaluation at 26 weeks' gestation. A male infant was delivered at 36.4 weeks of gestation via Cesarean section who developed progressive dyspnea shortly after birth. Magnetic resonance imaging (MRI) studies of his head showed the expansile, poorly demarcated mass in the pons with minimal heterogeneous enhancement and severe communicating hydrocephalus. Despite aggressive management, including dexamethasone treatment, the infant expired on the third day of postnatal life. On postmortem examination cut sections through the brainstem and cerebellum disclosed the neoplasm that infiltrated the entire pons, extended into the midbrain, medulla, cerebellar peduncles, and caudal diencephalon. Histological sections demonstrated an anaplastic oligodendroglioma infiltrating the pons, 4th ventricle, midbrain, medulla, cerebellar white matter, posterior thalamus, and occipital white matter. The pathological features of the lesion distinguish it from previous reports in which spontaneous regression of pontine gliomas occurred and argue in favor of establishing a tissue diagnosis to plan for aggressive versus conservative management.

## 1. Introduction

Congenital and perinatal primary brain neoplasms are extremely rare as the incidences range from 1 to 30 cases per million [1–4]. Brainstem neoplasms comprise 0.5%–1.5% of all brain tumors diagnosed during infancy [5]. The typically high-grade nature of perinatal and neonatal brainstem neoplasms harbors poor prognoses with survival rates of less than 2 years from diagnosis [1, 4]. Herein, we report an unusual case of congenital anaplastic oligodendroglioma that arose in the pons and was detected on in utero ultrasound (US) and magnetic resonance imaging (MRI) studies. The clinical and radiology aspects of this case have already been reported [6]. Herein, we are describing in detail the neuropathological findings and provide photographs of the gross and histopathological features of this rare tumor.

## 2. Report

**Figure 1:**
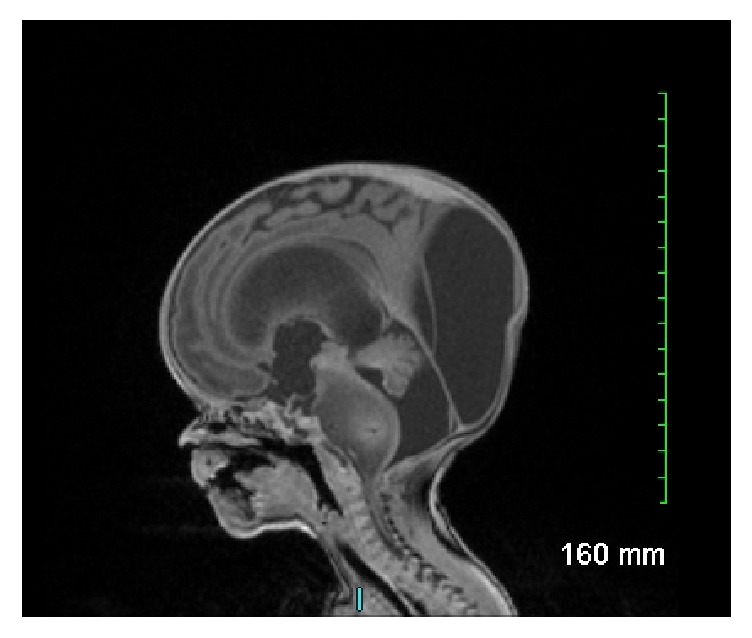
MRI showing an expansile, poorly demarcated mass in the pons with minimal heterogenous enhancement and severe communicating hydrocephalus.

A 33-year-old G6 P3 woman presented for prenatal evaluation at 26 weeks of gestation. Past medical history was noncontributory. Fetal US examination revealed findings consistent with macrocephaly, triventricular hydrocephalus, and small cerebellar hemispheres, but no evidence of spina bifida. Fetal MRI detected an expanding, poorly demarcated brainstem mass that was centered in the pons, extended into the midbrain, medulla, and cerebellar peduncles, and associated with dilated ventricles. A presumptive diagnosis of diffuse pontine glioma was made. Cesarean section at 36 weeks and 4 days produced a 3415-gram male infant who had Apgar scores of 7 and 9 at 1 and 5 minutes after birth, respectively. Head circumference was 41.2 cm (normal 33–36 cm). Shortly after birth, the infant developed progressive dyspnea. He was intubated and transferred to the neonatal intensive care unit. MRI studies of his head again showed the expansile, poorly demarcated mass in the pons with minimal heterogenous enhancement and severe communicating hydrocephalus (Figure 1). Neurosurgical intervention was sought for placement of an extraventricular drain (EVD) to reduce intracranial pressure and severity of respiratory distress. Despite aggressive management, including dexamethasone treatment, the infant expired on the third day of postnatal life.

## 3. Postmortem Examination

Postmortem examination revealed a full-term, well developed male infant with macrocephaly. Karyotype revealed normal male, 46 XY. The general autopsy findings showed large size for stated gestational age male neonate weighing 3239 g with expected weight of 2383 ± 373 g. Examination of the internal organs was unremarkable except for signs of moderate stress-induced thymic involution, as evident by cortical lymphocyte depletion on histologic examination.

**Figure 2:**
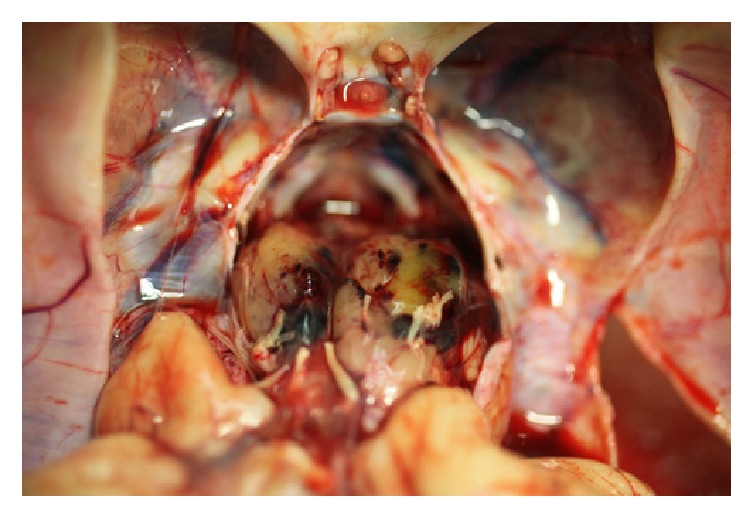
Upon opening the skull the brainstem was obscured by a large tumor with variegated appearance, soft somewhat gelatinous consistency, showing multiple hemorrhagic areas. (Brain is reflected posteriorly to expose the posterior cranial fossa; sella turcica with pituitary gland and transected optic nerves are seen at mid upper portion of the photograph.)

**Figure 3:**
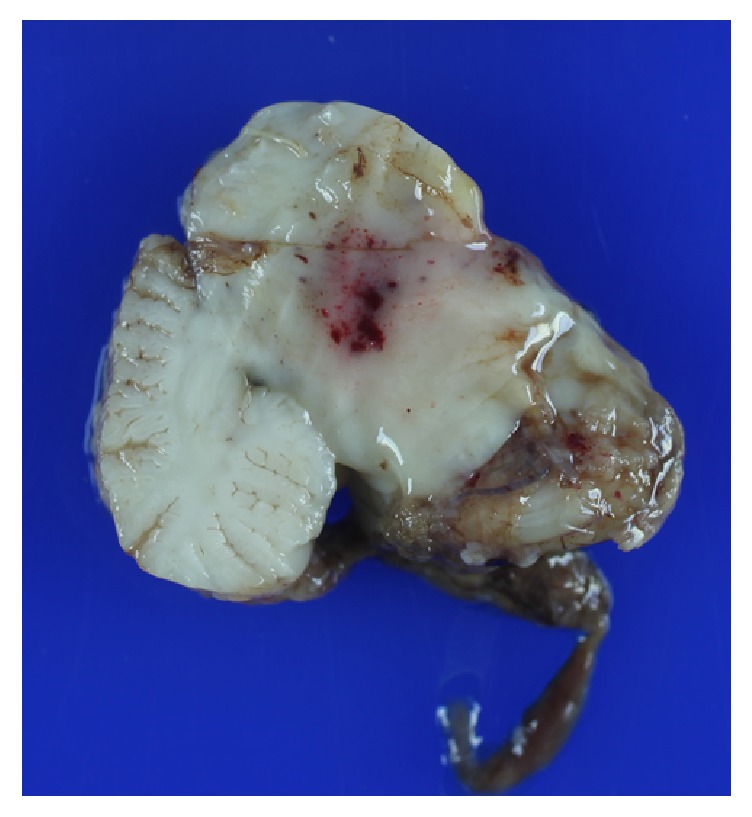
Cut sections through the brainstem and cerebellum showing the neoplasm infiltrating the entire pons, extending into the midbrain, medulla, cerebellar peduncles, and caudal diencephalon. Specimen after formalin fixation.

The fixed brain weighed 537 gms (normal 250–334). Small intra-arachnoid hemorrhages surrounded the ventrolateral surfaces of the brainstem and cerebellum and the right superior parietal region. The brain was diffusely swollen and had early encephalomalacia with pressure necrosis of all cranial nerves. In addition, severe hydrocephalus (3 to 5 times normal) involving the lateral and third ventricles resulted in extreme thinning of the cortex and compression of subcortical nuclei. Recent infarcts were present in the distribution of both middle and posterior cerebral arteries. The brainstem was occupied by a large tumor with variegated appearance and soft somewhat gelatinous consistency, showing multiple hemorrhagic areas (Figure 2). Cut sections through the brainstem and cerebellum disclosed the neoplasm that infiltrated the entire pons, extended into the midbrain, medulla, cerebellar peduncles, and caudal diencephalon (Figure 3). The presence of a large acute hemorrhage in the pons (2 × 2 × 2 cm) and cerebellum obstructed the aqueduct and 4th ventricle and was likely the cause of death.

**Figure 4:**
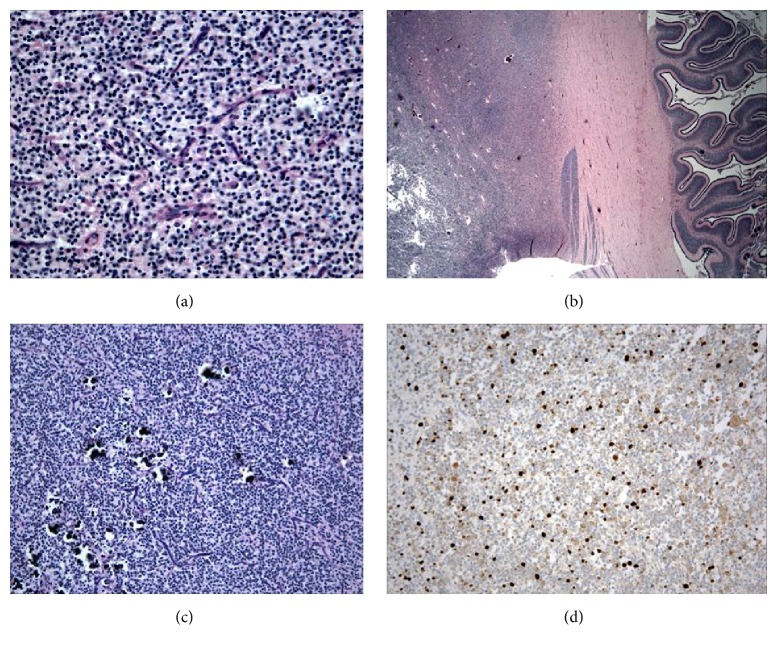
(a) The majority of the neoplasm showed cells with uniform, round nuclear morphology, diffuse chromatin, scant cytoplasm, and characteristic thin-walled chicken-wire vasculature. (b) Extensively infiltrative growth pattern of tumor permeating the pons, 4th ventricle, midbrain, medulla, cerebellar white matter, posterior thalamus, and occipital white matter. (c) Hypercellular areas with foci of calcifications. (d) Immunohistochemical staining demonstrated abundant labeling for Ki-67.

Histological sections stained with Luxol Fast Blue-Hematoxylin and Eosin demonstrated an anaplastic oligodendroglioma infiltrating the pons, 4th ventricle, midbrain, medulla, cerebellar white matter, posterior thalamus, and occipital white matter (Figure 4(b)). Cytologically, the majority of neoplastic cells had uniform, round nuclear morphology with diffuse chromatin, scant cytoplasm, scattered mitoses, foci of calcification (Figure 4(c)), and characteristic thin-walled chicken-wire vasculature (Figure 4(a)). In addition, small foci with astrocytic or neuronal (ganglion cell) features were also present. Higher grade areas of tumor (WHO Grade III/IV) were associated with foci of increased nuclear pleomorphism, necrosis, endothelial proliferation, and hemorrhage. Immunohistochemical staining demonstrated abundant labeling for Ki-67 (Figure 4(d)), scattered neoplastic cells with immunoreactivity for synaptophysin, NeuN, neuron-specific enolase, or glial fibrillary acidic protein (GFAP). Tumor cells were negative for p53 and myelin basic protein. Reactive cells associated with foci of necrosis were positive for CD68 (macrophages) or GFAP. The abundant Ki-67 labeling, together with hypercellularity, nuclear pleomorphism, necrosis, and extensively infiltrative growth pattern reflect aggressive behavior of the neoplasm, corresponding with the clinical course.

## 4. Discussion

Oligodendroglioma is the third most common glioma, accounting for 2–5% of primary brain tumors, 5–18% of all glial neoplasms, and less than 1% of pediatric central nervous system neoplasms. Prevalence rates are difficult to determine due to lack of standardized morphologic criteria and immunohistochemical or molecular biomarkers. Most oligodendrogliomas are diagnosed in adults, with peak incidences in the fourth or fifth decade. In adults, anaplastic oligodendrogliomas are detected later, with peak incidences in the sixth or seventh decade [7].

Oligodendroglial neoplasms in children arise 4–10 times more often in supratentorial brain structures, compared to infratentorial lesions [8]. Cerebellar oligodendrogliomas account for only 3% of the cases and brainstem and spinal cord only 1%. Other rare sites include leptomeninges (oligodendrogliomatosis), cerebellopontine angle, cerebral ventricles, retina, and optic nerve. Brainstem gliomas in children range widely in histologic grade or clinical behaviors. Diffuse intrinsic pontine glioma (DIPG) accounts for approximately 80% of pediatric brainstem gliomas [8].

The prognostic significance of brain tumor classifications and grading has been difficult to evaluate due to periodic updating, changing grading criteria, and low interrater reproducibility even among specialized pediatric pathologists. The most widely used histological classification system of brain tumors is that of the World Health Organization (WHO). However, classifying high-grade gliomas by histological features can be challenging, leading to limited reproducibility and significant interobserver variability. In addition, despite similar histological terminology, adult and pediatric high-grade gliomas differ in terms of prognosis [9].

An alternative classification system appears to be more practical. The Sainte-Anne (SA) classification has been reported as a simpler and more standardized grading system with more reproducibility than the WHO system. It encompasses more tumor grades or subtypes, oligodendroglioma grades A and B, as well as the malignant glioneuronal tumor (MGNT).

It is highly unusual for oligodendrogliomas to be diagnosed in children. The peak incidence is between 6 and 12 years of age. In addition, pediatric oligodendrogliomas appear to be genetically distinct from tumors of adults, rarely exhibiting 1p/19q codeletions.

Oligodendrogliomas are even more rarely seen in fetuses comprising just 0.5–1.5% of brain tumors diagnosed during infancy [4]. The present case is extremely unusual because it was detected by fetal US and MRI and therefore developed in utero. The extremely short survival is characteristic of the disease since survival is less than 18–24 months for the vast majority of children with primary brainstem neoplasms [4, 10]

Several reports have been published describing expansile brainstem mass lesions in neonates, in which the typical clinical and radiographic findings of diffuse glioma resolved spontaneously [2, 10–12]. Unfortunately, most of these cases failed to provide supportive histopathologic diagnoses, making it difficult to know whether such lesions can “self-cure.” There was one report of a histopathologically proven case of pontine glioma that resolved spontaneously [12]. Reports of spontaneous remissions of brainstem tumors have led clinicians to use conservative therapeutic approaches such as placement of a ventricular drain to relieve hydrocephalus. Unfortunately, for the present case, detailed clinical and radiology presentation which has been described in a separate report [6], the clinical deterioration was very rapid and the child ultimately died from brainstem hemorrhage and respiratory arrest.

## 5. Conclusions

We present an unusual and rare example of an anaplastic pontine oligodendroglioma arising in a fetus that was detected by fetal US and MRI and confirmed by neonatal US/MRI and autopsy. The extensive degree to which the neoplasm infiltrated the brainstem, destroying major respiratory and autonomic centers, together with its aggressive growth leading to obstructive hydrocephalus, respiratory failure, and hemorrhage accounts for the malignant clinical course. The pathological features of the lesion distinguish it from previous reports in which spontaneous regression of pontine gliomas occurred and argue in favor of establishing a tissue diagnosis to plan for aggressive versus conservative management.
